# Bonding Properties of Embedded Fiber Reinforced Polymer Strip-Engineered Cementitious Composite Joints

**DOI:** 10.3390/polym17081049

**Published:** 2025-04-12

**Authors:** Weiwen Li, Wujun Fang, Yao Lu, Wanye Li, Jingming Yang, Hao Wang, Peng Wang, Yaocheng Wang, Hongzhi Cui

**Affiliations:** 1 Guangdong Provincial Key Laboratory of Durability for Marine Civil Engineering, College of Civil and Transportation Engineering, Shenzhen University, Shenzhen 518060, China; liweiwen@szu.edu.cn (W.L.); fangwujun@cribc.com (W.F.); liwanye2022@email.szu.edu.cn (W.L.); wangyc_szu@126.com (Y.W.); h.z.cui@szu.edu.cn (H.C.); 2 China National Key Laboratory of Green and Long-Life Road Engineering in Extreme Environment (Shenzhen), Shenzhen 518060, China; 3 MCC Group, Central Research Institute of Building and Construction (Shenzhen) Co., Ltd., Shenzhen 518055, China; yangjingming@cribc.com; 4 Southern Company of China Construction Eighth Engineering Division Co., Ltd., Shenzhen 518000, China; 18585804669@163.com

**Keywords:** fiber reinforced polymer (FRP), engineered cementitious composite (ECC), pull-out behavior, embedded length, trilinear bond–slip relationship

## Abstract

The combination of fiber reinforced polymer (FRP) and engineered cementitious composite (ECC) has emerged as a promising method for strengthening reinforced concrete (RC) structures. By embedding FRP within an ECC to form a composite reinforcement layer, the advantages of both materials can be effectively harnessed, and the dense ECC matrix can be employed to safeguard FRP from adverse environments. Significantly, the interface bonding property constitutes the key for the two materials to collaborate effectively. In light of the research gap related to the bonding performance of embedded FRP strips in ECC joints, this study conducted a bench-scale investigation into the pull-out behavior of carbon FRP (CFRP) strips within an ECC. The relationship between the average bonding strength (2.84 MPa~4.77 MPa) and the embedded length of FRP strips was established. Additionally, the pull-out mechanism of FRP strips within an ECC matrix was utilized to elucidate the influence of the embedded length on the distinct behavior of FRP strips within an ECC. An analytical method for predicting the full-range behavior of embedded FRP strip–ECC joints by using a trilinear bond–slip relationship was introduced. Four key parameters of the trilinear bond–slip relationship for embedded FRP strip–ECC joints were provided to meet the requirements of future engineering applications.

## 1. Introduction

Fiber reinforced polymer (FRP), which is made up of fibers and epoxy resin, has received significant attention in civil engineering for its application in structural reinforcement. This material is highly valued in both practical projects and academic studies because of its lightweight nature, excellent strength, and resistance to corrosion [1,2,3,4,5]. FRP is typically employed to strengthen the structure in the form of external bonds to prevent further deterioration of the structure’s performance [6,7,8,9,10]. Nevertheless, as research progresses, scholars have discovered that the interfacial bonding properties of FRP concrete will deteriorate under high temperature and exposure to moist or dry/wet cycling [11,12,13,14,15]. For example, when the temperature increases from 20 °C to 300 °C, the FRP’s bond strength reduces by between 80 and 90% [13], and defects as well as loose microstructure form in both FRP and concrete under dry/wet cycling condition (more than 50 cycles) [14]. To overcome these issues, scholars have employed inorganic matrices to substitute organic resins as the interface medium between FRP and concrete. Among them, engineered cementitious composite (ECC), as an inorganic material, is a research focus [16,17,18,19,20,21,22,23]. This is because ECC possesses numerous advantages, such as enhanced toughness, a multiple and tight cracking nature, and excellent tensile ductility, surpassing ordinary Portland cement (OPC) paste, mortar, and concrete. It exhibits ductility in the hardened state and flexibility in the fresh state [24,25]. ECC demonstrates enhanced durability in various environmental conditions, featuring a low permeability coefficient and greater resistance to steel corrosion than traditional concrete. Previous studies [26,27,28] have suggested that ECC could achieve minor crack widths (typically less than 100 µm) during the multi-cracking stage and significant tensile strain (usually ranging from 3% to 5%), and is suitable for developing energy-absorbing structures and applications in adverse environmental conditions.

**Figure 1:**
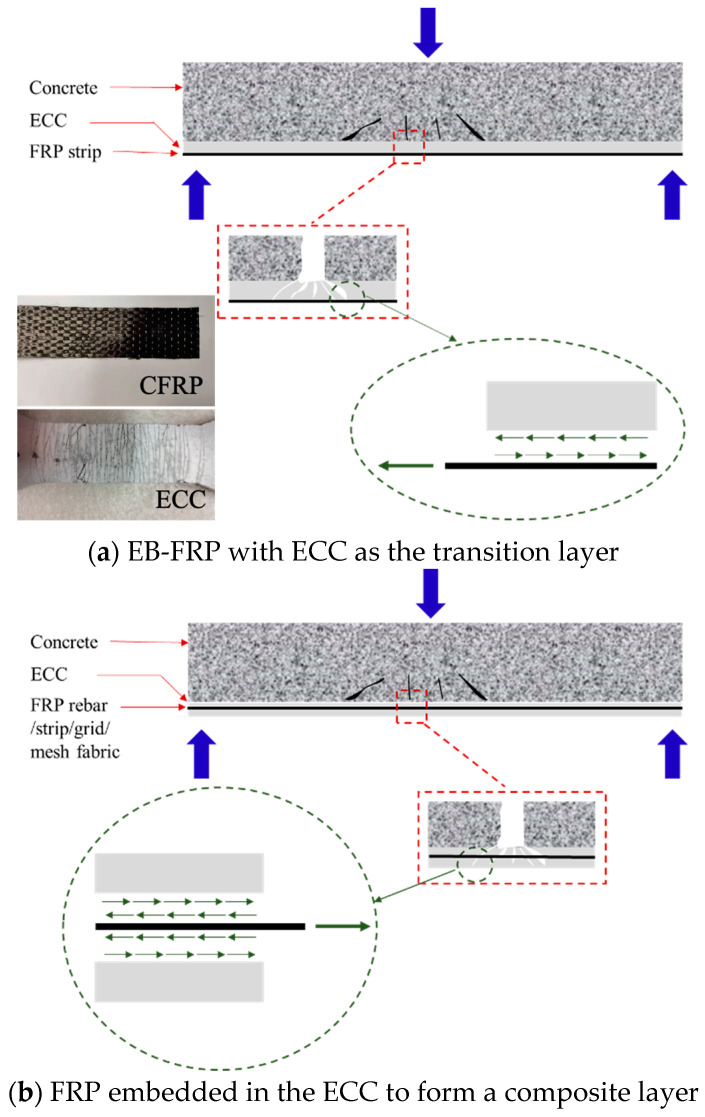
Enhancing performance of structural members with FRP-ECC composites.

As a protective or enhancement layer, the combination of FRP and ECC can be classified into two types: one is the externally-bonded FRP (EB-FRP) with ECC serving as the transition layer [16,29,30], and the other is FRP embedded in the ECC to form a composite layer [31,32,33,34,35,36], as depicted in Figure 1. Academic research related to EB-FRP and the employment of ECC as a transition layer between FRP and concrete for strengthening the structure is ongoing [29,30]. As shown in Figure 1a, FRP is typically bonded on the surface of the member to be reinforced in the form of strips to restrain the expansion of cracks on the member surface. Zhou et al. [29] investigated the effect of ECC with different thicknesses on enhancing the EB-FRP strengthening efficacy. Their findings indicated that ECC significantly mitigated or eliminated the premature debonding of EB-FRP, which in turn significantly increased the member’s flexural capacity, ductility and energy consumption capacity compared to the conventional EB-FRP strengthened system. Afefy et al. [30] also drew a similar conclusion regarding the research results related to an enhancement of the flexural behavior of FRP-strengthened reinforced concrete (RC) beams using ECCs as the transition layer. They all clearly indicated that the interfacial bonding performance was a key factor for the strengthening effectiveness. Sui et al. [16] studied the influence of ECC on the interface performance of FRP concrete and pointed out that the thickness of ECC has a significant impact on the transmission of interface shear stress.

Figure 1b presents the alternative approach of embedding different types of FRP (such as rebar, strip, grid, or mesh fabric) within the ECC to form composite layers. Different from the manner of forming EB-FRP with ECC as the transition layer, this method can leverage the dense property of ECC to protect the FRP from the detrimental effects of the harsh external environment. Guo et al. [32] studied the shear strengthening effect on RC beams by utilizing an FRP grid and ECC. The experimental results demonstrated that the FRP grid in the ECC layer effectively enhanced the shear strength of the RC beam; however, the higher stiffness of the composite layer caused the debonding of the FRP grid–ECC composite layer. The stiffness of an FRP mesh fabric is lower than that of an FRP grid. 

Li et al. [17] employed a composite plate formed by FRP mesh fabric and sulfoaluminate cement ECC (SAC-ECC) to conduct bending reinforcement tests on RC beams and discovered that the debonding failure of the composite plate was not obvious. Jiang et al. [35] designed pull-out tests to investigate the bond properties between the FRP mesh fabric and ECC, considering the short fiber effect. The results showed that stress transfer from the FRP mesh fabric to the matrix was achieved through the bond properties, which affected the tensile behaviors of the FRP mesh fabric–ECC composite layer.

In summary, regardless of which combination of the aforementioned two approaches is employed, the bonding properties of the interface between different materials constitutes an important research topic [37]. The interfacial strength between the FRP-ECC composite layer and the concrete determines the strengthening efficiency, while the interfacial performance of FRP and ECC determines the utilization rate of the composite layer. Regarding the bonding properties of embedded FRP and ECC, there are preliminary research findings on the bonding properties between FRP (mesh fabrics [35], grids [38] and rebar [39]) and ECC. Jiang et al. [35] observed three failure modes in an FRP mesh fabric pull-out experiment in an ECC, namely textile rupture, pulling out, and a compound mode with both pulling out and textile rupture. Different from FRP mesh fabrics, the pull out test results of Deng et al. [38] indicated that FRP grids had two failure modes, grid rupture and pulling out, but no compound failure mode with both pulling out and grid rupture. In addition, Zhao et al. [39] found that the pulling out of large diameter FRP bars in ECC would cause splitting failure of an ECC matrix, and a size effect was obvious. However, the pull-out behavior of embedded FRP strips in ECC has not been extensively studied.

Consequently, a series of pull-out tests were conducted in this study to elucidate the bonding behaviors between embedded FRP strips and ECC. The influence of different embedded lengths on pull-out behavior was investigated. The quantitative relationship between average shear stress and embedded length was established, and the analytical solution of a trilinear bond–slip relationship of embedded FRP strip–ECC joints was obtained by employing analytical expressions. This study can serve as a reference for the application of FRP strips in ECC composite members in practical engineering.

## 2. Experimental Program

### 2.1. Material Properties

As the standard bonding matrix, the ECC was prepared using a fixed mix-proportion developed by Li et al. [17], as shown in Table 1. The polyethylene (PE) fiber utilized is manufactured in China, with a diameter of 20 μm and length of 18 mm. Table 2 and Table 3, respectively, list the chemical compositions of the fly ash and cement, as well as the physical and mechanical properties of the PE fibers. The quartz sand used in this experiment has a maximum particle size of 180 μm and an average particle size of 135 μm. In this experiment, polycarboxylic acid superplasticizer with a water reduction rate of 41% was employed, which is produced by Xiamen Kezhijie New Materials Group Co., Ltd. (Xiamen, China).

**Figure 2:**
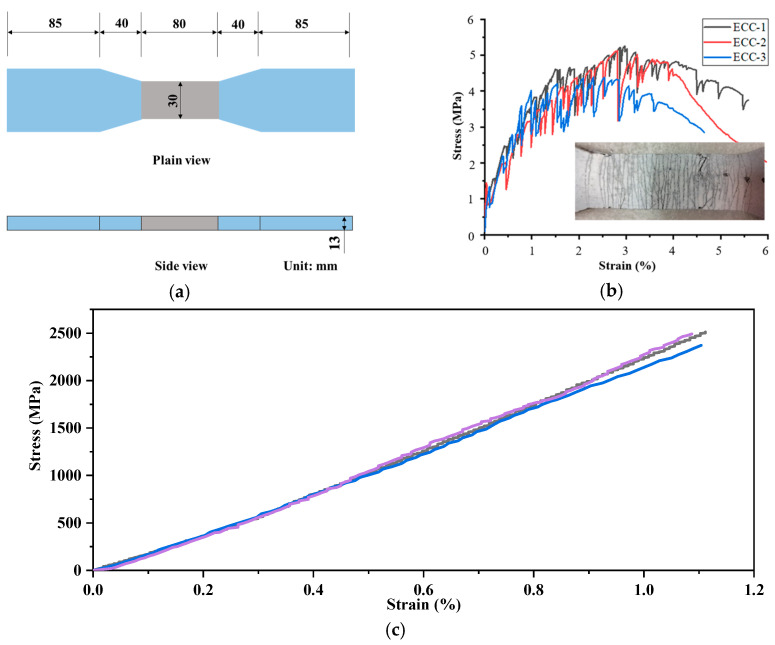
Properties of ECC and FRP: (**a**) the dog bone-shaped sample of ECC, (**b**) stress–strain curve and failure image of ECC and (**c**) stress–strain curve of FRP (Adapted from [36], Elsevier, 2023).

The preparation of all ECC mixtures was carried out using a 12 L shear-type Hobart mixer. Initially, the solid components, such as cement, silica fume, fly ash, silica sand, and filler materials, were dry-mixed for 3 min. Following this, water and superplasticizer were introduced into the mixture and blended for an additional 3 min. Lastly, fibers were gradually incorporated into the fresh mortar and mixed for 5 more minutes until no fiber agglomeration was visible. Once the mixing process was complete, the resulting ECC mixtures were poured into molds. The specimens were then removed from the molds after 24 h and cured under ambient conditions of 25 ± 3 °C temperature and 50 ± 5% relative humidity until they were tested after 28 days of curing. As shown in Figure 2a, a dog bone-shaped specimen was used to evaluate the uniaxial tensile properties of the matrix in accordance with JSCE-2008 [40]. An extensometer was utilized to measure the deformation over an 80 mm gauge length. Figure 2b presents the stress–strain behavior of ECC, exhibiting apparent strain-hardening behavior and multiple cracking after the tensile stress reached about 1.4 MPa. The 28-day cubic standard compressive strength of concrete is 40.8 MPa.

The carbon FRP (CFRP) strips employed in this study were produced by Carben Technology Group Co., Ltd. (Tianjin, China). Epoxy resin (the ultimate tensile stress and strain are 38 MPa and 1.5%, respectively) was utilized to impregnate the FRP sheets, and upon curing, the FRP sheets were cut to obtain the FRP strips required for the test. The mechanical properties of the FRPs used were obtained through uniaxial tensile testing at a constant strain rate of 1 mm/min, according to the standard GB/T 3354-2014 [41]. Figure 2c and Table 4 present the mechanical and geometrical properties of the CFRP strips.

### 2.2. Specimen and Test Setup

**Figure 3:**
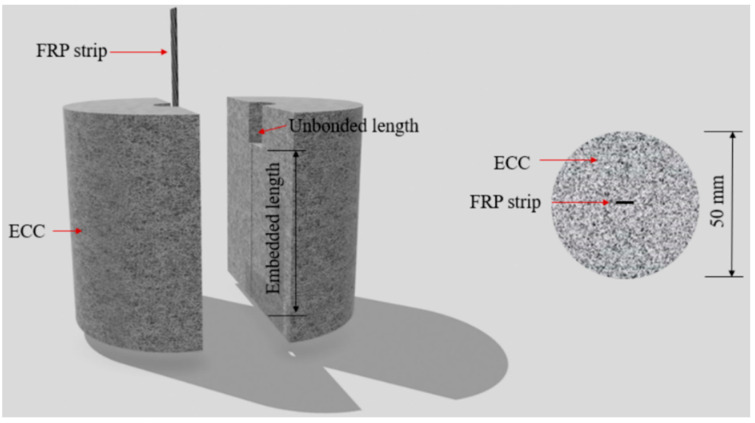
Schematic illustration of the specimen.

**Figure 4:**
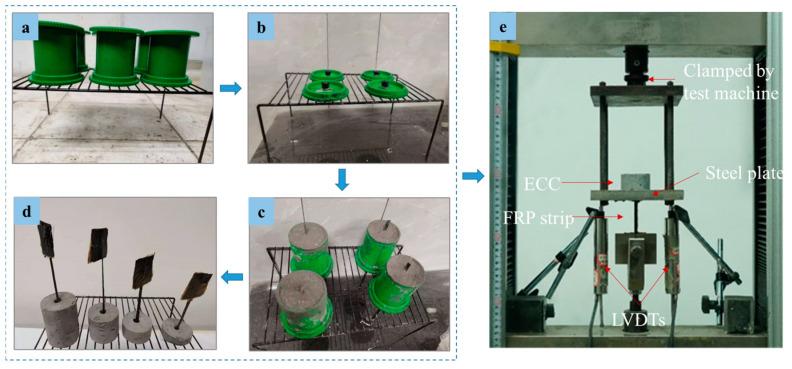
Sample preparation and setups for testing: (**a**) brush the mold with oil, (**b**) reserve the unbonded area, (**c**) ECC casting, (**d**) obtained specimens and (**e**) pull-out tests.

Based on the previously used central pull-out specimens [42,43], a novel pull-out specimen for the FRP strip–concrete interface has been developed, as illustrated in Figure 3. The specimen consists of a centrally embedded FRP strip and an inner ECC cylinder with an inner diameter of 50 mm. To prevent the ECC from experiencing local end failure resulting from stress concentration, an unbonded region (with a length of 10 mm) is set by isolating the FRP strip from the ECC via polyvinyl alcohol (PVA) tubes with an inner diameter of 10 mm. For the bonded region of the FRP strip–ECC interface, different embedded lengths are set. The pull-out specimens are prepared in accordance with the manufacturing flow in Figure 4a–d. Firstly, after cleaning the mold, a coat of lubricating oil is applied inside (Figure 4a). Then, the FRP strip and PVA tubes are fixed to the mold (Figure 4b) and the evenly stirred ECC is cast into the mold (Figure 4c). Following 24 h of air curing, the specimens are removed from their molds and subsequently subjected to moist curing at room temperature with a relative humidity of 95% for 27 days (Figure 4d). Finally, the loading end of the FRP strip is strengthened to prevent stress concentration failure in the loading section. The specimens are numbered in the form CN, where C denotes CFRP and N denotes the embedded lengths of the FRP strips (unit: mm). For instance, a specimen named C30 indicates the use of CFRP and an embedded length of 30 mm. The detailed experimental parameters of the specimens are listed in Table 5.

The pull-out tests were conducted with a 300 kN MTS testing machine produced by Dongguan Pengsheng instrument Equipment Co., Ltd, Dongguan, China, as shown in Figure 4e. The test specimen is placed on a special reaction frame, which is connected to the test machine. The steel plate of the reaction frame has a reserved hole with a length of 50 mm and a width of 10 mm to allow the FRP strip through the hole. The matrix is in the middle of the reaction frame and is kept in line with the FRP strip and the lower fixture to avoid eccentricity. The test speed is controlled by displacement and the rate is 1 mm/min. Two linear variable differential transformers (LVDTs), one end of which is fixed on the beam of the testing machine by magnetic support and the other end is placed on the reaction frame, are used. The covariance of both LVDTs measurements is 1.3%. The slip between the FRP strip and the ECC matrix at the loading end is determined by subtracting the deformation of the FRP strip exposed outside the ECC from the displacement measured by the two LVDTs [44].

(1) $$S=∆-\frac{Fl}{EA}$$

where S is the slip between the FRP strip and ECC matrix, ∆ is the displacement measured by the two LVDTs, F is the load borne by the FRP strip, l is the length of the FRP strip exposed outside the ECC, and E and A represent the elastic modulus and cross-sectional area of the FRP strip, respectively.

## 3. Experimental Observation

**Figure 5:**
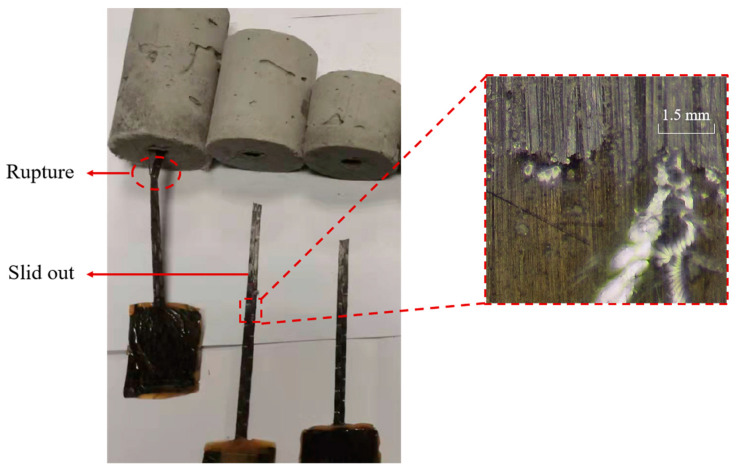
Failure modes.

As shown in Figure 5, the pull-out specimens undergo two failure modes: Slide-out failure and FRP strip rupture failure. When the embedded length of the FRP strip does not exceed 50 mm, the specimen will fail to slide out. It can be observed that after the FRP strip is pulled out of the ECC matrix, the epoxy resin impregnated on the FRP surface is scratched, and part of the epoxy resin remains in the ECC matrix. This means that the interface failure of the FRP strip–ECC joints is caused by the interlayer shear failure of the epoxy resin and the FRP surface. This is different from the phenomenon that the epoxy resin has good bonding with the FRP plate as observed by Lin et al. [42] in the pull-out test of the FRP plate in the confined concrete. This may be due to the fact that the ECC’s matrix is relatively dense and has a higher strength than concrete, resulting in better interfacial strength between the epoxy resin and ECC than between the epoxy resin and concrete. FRP strip rupture failure refers to the failure mode where the embedded length is relatively long (the embedded length is 70 mm).

**Figure 6:**
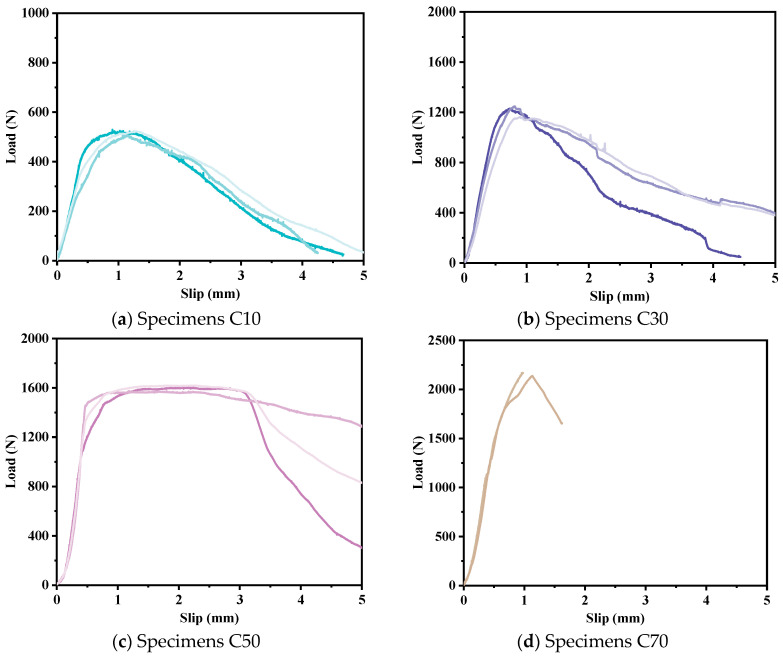
Typical load–slip curves for specimens.

Figure 6 shows the typical load–slip curve of the pull-out specimens, showing three behaviors. The pull-out load of specimens with an embedded length of 70 mm (C70) is the largest. After reaching the peak load, the load–slip curve abruptly drops due to the rupture of the FRP strips, and the experimental process is forced to stop. On the contrary, the load corresponding to FRP slide-out failure (specimens C50, C30 and C10) decreases slightly with unit slip after reaching the peak value. It is worth noting that the load–slip curves of specimens C30 and C10 show similar behavior, and the rate of load decline changes from fast to slow after the load reaches its peak. However, the load–slip curve of specimen C50 has a long similar plateau phase, which is different from the pull-out behavior of specimens C30 and C10. In addition, the post-peaking behavior [23] (residual bond strength hardening) observed in the pull-out behavior of FRP bars in ECC is not evident in the load–slip curve of the FRP strips. This is attributed to the absence of ribs on the FRP strips, which are present on FRP bars.

**Figure 7:**
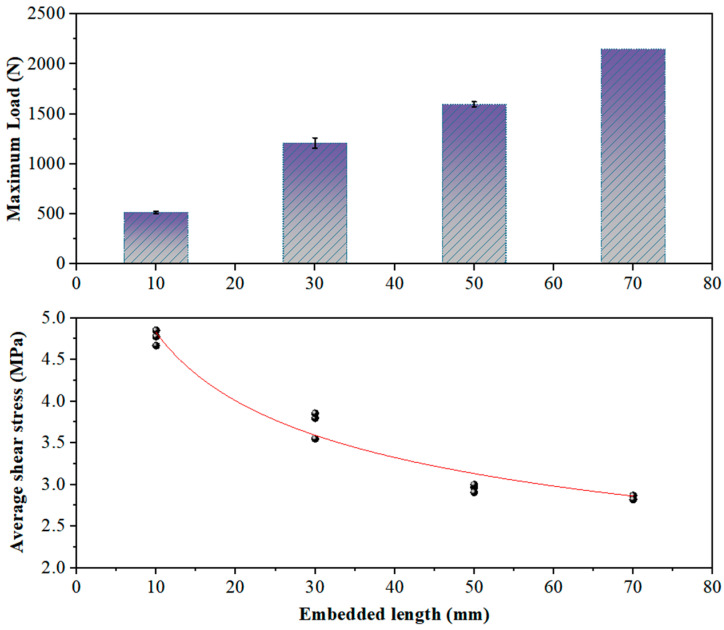
Effect of embedded length on load and average shear stress.

While it remains challenging to measure and predict the distribution of bond stress along the embedded length, the average shear stress along this length can still be used to evaluate the bond efficiency as influenced by various factors, as has been conducted in other studies [35]. As can be seen from Figure 7, although the pull-out load increases with the increase of the embedded length, the average shear stress decreases with the increase of the embedded length of the FRP strips. This is because the actual bond stress changes along the bonding length. When the embedded length of the FRP strips is longer, the bond shear stress distribution is more uneven. The greater the embedded length, the lower the average bonding force per unit area. Figure 7 also shows the fitting of the average shear stress of FRP strips at different embedded lengths obtained by the test. A power function is used to fit the relationship between the average shear stress and the embedded length. The specific form of the fitting formula is presented in Equation (2).

(2) $$y=ax^{m}$$

The a can be considered as a parameter related to the physical properties of the FRP (in particular, the elastic modulus of the FRP), and m can be considered as a correction factor for the embedded length. After fitting, the formula of the relationship between adhesive stress and the length of different fibers is obtained. In the case of CFRP strips, a= 8.96 and m= −0.27 ($R^{2}=\sim$0.955).

## 4. Analytical Extraction of the Bond–Slip Relationship for the FRP Strip–ECC Joints

### 4.1. Trilinear Bond–Slip Relationship

**Figure 8:**
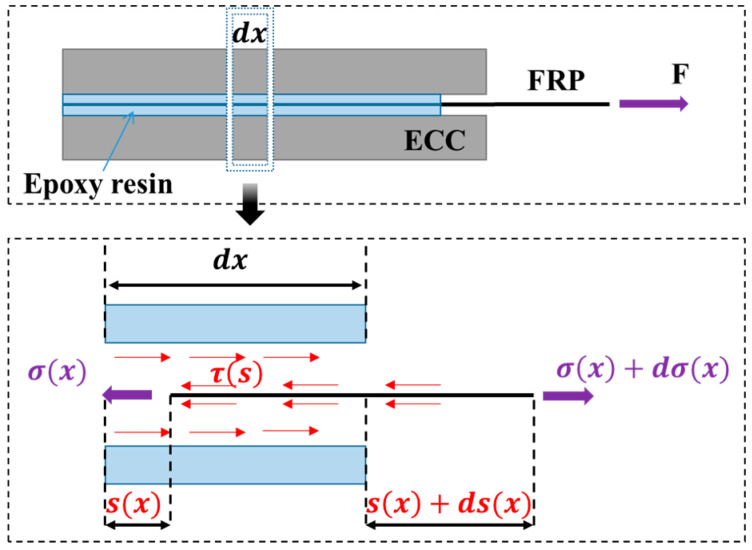
Schematic side view of a single-lap direct-shear test.

The process of pull-out of an FRP strip from ECC involves the transfer of shear stress at the two interfaces between the FRP strip and ECC, as shown in Figure 8. Intercepting a microunit at x position, it is not difficult to find that the relationship between the tensile stress element of FRP strip at x position and the shear stress at the interface can be expressed as follows:

(3) $$d\sigma \left(x\right)\times t=2\times \tau \left(s\right)\times dx$$

Substituting $\sigma \left(x\right)=E\times \varepsilon \left(x\right)=E\times \frac{ds(x)}{dx}$ into Equation (3) yields the following equation:

(4) $$\tau \left(s\right)=\frac{E\times t}{2}\times \frac{d\varepsilon (x)}{dx}=\frac{E\times t}{2}\times \frac{d^{2}s(x)}{dx^{2}}$$

where E is the elastic modulus of the FRP strip, t is the thickness of the FRP strip, and $\sigma \left(x\right)$ and ε(x) represent the tensile stress and strain of the FRP strip, respectively. The physical implication expressed in Equation (4) is that the change in the tensile strain of the FRP strip at the x position is related to shear stress, $\tau \left(s\right)$, at the interface between the FRP strip and the ECC.

**Figure 9:**
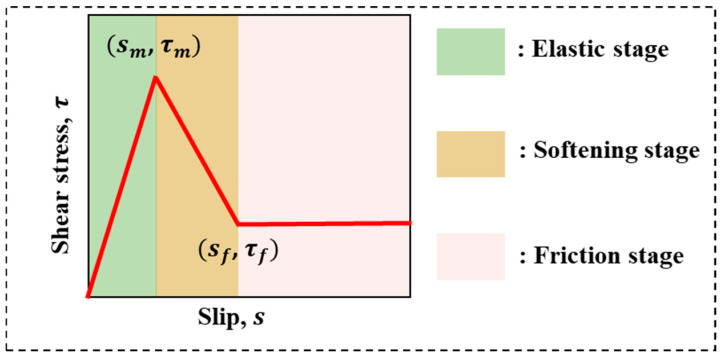
Trilinear bond–slip relationship of embedded FRP strip–ECC joints.

In fact, the shear stress $\tau \left(s\right)$ at x position is a function of the slip s(x). This study used a trilinear bond–slip relationship (Figure 9) [45,46] consisting of a linear elastic branch, a softening branch, and a friction branch to represent this function, which has been effectively verified in the studies [45,47,48] related to the interface of the two media. This implies that the nature of the pull-out behavior of the FRP strip in ECC is the dynamic equilibrium process of the integration of the pull-out load borne by the FRP strip and the shear stress (in these three distinct stages) distributed on the bond length of the interface between the FRP strip and ECC. During the entire pull-out process, the shear stress at the x position firstly increases from (s=0, τ=0) to ($s=s_{m}$, $\tau =\tau_{m}$) in the linear elastic stage; after a peak, it decreases from ($s=s_{m}$, $\tau =\tau_{m}$) to ($s=s_{f}$, $\tau =\tau_{f}$) at the softening stage, and then it enters the friction stage. The trilinear $\tau \left(s\right)$ is defined as:

(5) $$\tau \left(s\right)=\left\{\begin{array}{c} \frac{\tau_{m}}{s_{m}}s,\;\;\;\;\;\;\;\;\;\;0\leq s\leq s_{m}\\ \tau_{m}\left[\frac{\left(s_{f}-s\right)+\frac{\tau_{f}}{\tau_{m}}(s-s_{m})}{s_{f}-s_{m}}\right],\;\;\;\;\;\;\;\;\;\;s_{m}\leq s\leq s_{f}\\ \tau_{f},\;\;\;\;\;\;\;\;\;\;s\geq s_{f} \end{array}\right.$$

Substituting Equation (5) into Equation (4), a governing differential equation can be constructed. By utilizing the trilinear bond–slip relationship and boundary conditions, a solution to the interfacial governing equation can be derived. The outcome of the analytical derivation can be represented through the applied load–slip relationship at the loaded end (F-S relationship). A similar derivation process can also be found in the work of Zou et al. [45].

### 4.2. Analysis of the Full-Range Slid-Out Behavior of FRP Strips in ECC

As mentioned above, although specimens C50, C30 and C10 all exhibit slip-out failures, the load–slip response of C50 differs from that of C30 and C10, apparently owing to the varying embedded lengths. This section explains the full-range slide-out behavior of FRP in ECC, as well as the influence of the embedded length on the pull-out behavior. Since the trilinear bond–slip relationship is a piecewise function, there are different expressions for the solution based on the slip distribution and F-S relationship. Depending on the slip distribution, different bond–slip relationships or their combinations may occur along the bond length direction, resulting in different interface states.

**Figure 10:**
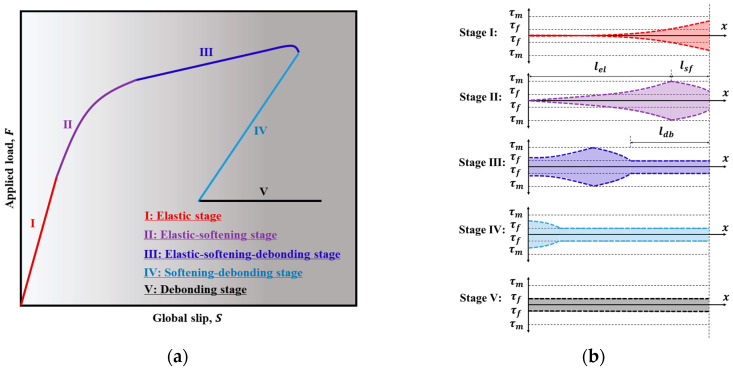
Evolution of *F*-*S* relationship and interfacial shear stress distribution for long embedded length: (**a**) the *F*-*S* relationship and (**b**) the interfacial shear stress distribution.

The F-S relationship and shear stress distribution along the interface refers to the long-embedded length case, as is reported in Figure 10. For long-embedded length, the typical F-S relationship is shown in Figure 10a. The F-S relationship response is divided into five stages, and the corresponding shear stress distribution along the interface is shown in Figure 10b.

When the slip at the loaded end, S, is less than $s_{m}$, and the free end slips, $s_{F}$, increase from zero to a small value, the shear distributed over the entire embedded length is governed by the first piece of Equation (5) (see stage I in Figure 10). The F-S relationship can be obtained as:

(6) $$P=\left(\frac{b\tau_{m}}{\lambda_{1}}\right)\frac{\tanh(\lambda_{1}L)}{s_{m}}$$

Equation (6) indicates that P increases proportionally with S. If $S=s_{m}$, interfacial softening initiates at the loaded end. With increasing S, part of the interface enters the softening stage, the rest remains in the elastic stage and the shear stress is distributed along the interface in stage II (as shown in Figure 10b). The softening part is denoted $l_{sf}$, while the elastic part is denoted by $l_{el}$. The associated load displacement curves are described and shown in stage II (as show in Figure 10a). The F-S relationship can be obtained from Equations (7)–(10).

(7) $$P=\frac{b\tau_{m}}{\lambda_{3}}\left[\frac{\lambda_{3}}{\lambda_{1}}\cos\left(\lambda_{3}l_{sf}\right)\tanh\left(\lambda_{1}l_{el}\right)+\sin(\lambda_{3}l_{sf})\right]$$

(8) $$S=(s_{f}-s_{m})\left[\frac{\lambda_{2}\sin\left(\lambda_{3}l_{sf}\right)\tanh(\lambda_{1}l_{el})}{\lambda_{1}\sqrt{1-\mu }}-\frac{\cos\left(\lambda_{3}l_{sf}\right)}{1-\mu }+\frac{s_{f}-\mu s_{m}}{\left(1-\mu )(s_{f}-s_{m}\right)}\right]$$

(9) $$L=l_{el}+l_{sf}$$

(10) $$l_{el}=\frac{1}{\lambda_{1}}\mathrm{arccosh}(\frac{s_{m}}{s_{F}})$$

At the end of this stage, part of the interface enters the debonding stage (the debonding part is denoted by $l_{db}$), while the rest is still in the elastic–softening stage. The F-S curve is shown in stage III of Figure 10a. Note that at this stage, the applied force is not constant but increases due to the phenomenon of friction. The F-S relationship can be obtained from Equations (10)–(14).

(11) $$P=b\tau_{m}\left\{\mu l_{db}+\frac{\sin(\lambda_{3}l_{sf})}{\lambda_{3}}+\frac{1}{\lambda_{1}}\cos\left(\lambda_{3}l_{sf}\right)tanh\left[\lambda_{1}(L-l_{sf}-l_{db})\right]\right\}$$

(12) $$S=s_{f}+s_{f}\lambda^{2}l_{db}\left[\frac{\mu l_{db}}{2}+\frac{\sin(\lambda_{3}l_{sf})}{\lambda_{3}}+\frac{1}{\lambda_{1}}\cos\left(\lambda_{3}l_{sf}\right)\tanh(\lambda_{1}l_{el})\right]$$

(13) $$L=l_{el}+l_{sf}+l_{db}$$

(14) $$l_{sf}=\frac{1}{\lambda_{3}}arccos\left[\frac{\mu +\frac{\lambda_{3}}{\lambda_{1}}\sqrt{1-{\left(\frac{s_{F}}{s_{m}}\right)}^{2}}\sqrt{2-\mu^{2}-{\left(\frac{s_{F}}{s_{m}}\right)}^{2}}}{2-{\left(\frac{s_{F}}{s_{m}}\right)}^{2}}\right]$$

When the slip at any location of the interface exceeds $s_{m}$ with a value of S that is larger than $s_{f}$, there is no elastic stage at the interface, and the shear stress distribution is shown in Figure 10b, stage IV. The corresponding F-S curve is shown in stage IV in Figure 10a, accompanied by snap-back phenomenon [49]. The F-S relationship can be obtained from Equations (15)–(18).

(15) $$P=\mu \tau_{m}b\left[L-l_{sf}+\frac{\tan(\lambda_{3}l_{sf})}{\lambda_{3}}\right]$$

(16) $$P=\mu \tau_{m}b\left[L-l_{sf}+\frac{\tan(\lambda_{3}l_{sf})}{\lambda_{3}}\right]$$

(17) $$L=l_{sf}+l_{db}$$

(18) $$l_{sf}=arccos\left[\left(\frac{\mu }{1-\mu }\right)\frac{s_{f}-s_{m}}{\left(\frac{s_{f}-\mu s_{m}}{1-\mu }-s_{F}\right)}\right]$$

When the slip exceeds $s_{f}$ at any location along the interface, the shear stress is equal to the frictional stress along the entire interface. The corresponding F-S curve is shown in stage V of Figure 10a, and the shear stress distribution is shown in Figure 10b stage V. The pull-out load is given as:

(19) $$P=2b\mu \tau_{m}L$$

where $\mu =\frac{\tau_{f}}{\tau_{m}}$, $\lambda =\sqrt{\frac{\tau_{m}}{s_{f}Et}}$, $\lambda_{1}=\lambda \sqrt{\frac{2s_{f}}{s_{m}}}$, $\lambda_{2}=\lambda \sqrt{\frac{2s_{f}}{s_{f}-s_{m}}}$, $\lambda_{3}=\lambda_{2}\sqrt{1-\mu }$.

**Figure 11:**
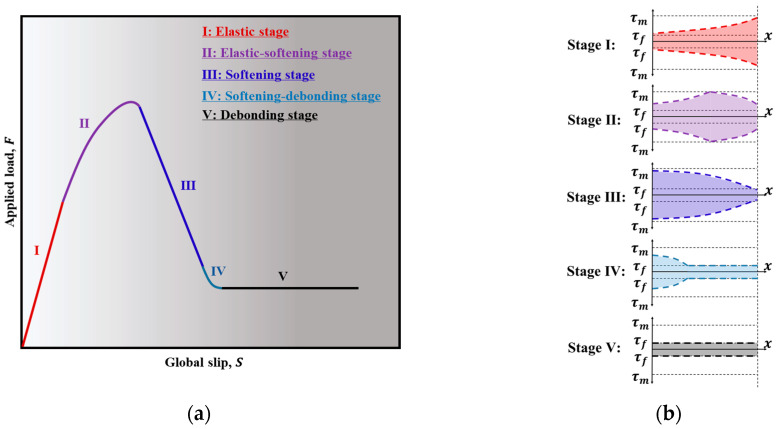
Evolution of *F*-*S* relationship and interfacial shear stress distribution for the embedded length less than the fully established softening length: (**a**) the *F*-*S* relationship and (**b**) the interfacial shear stress distribution.

Figure 11 illustrates the F-S relationship and the distribution of shear stress along the interface, which corresponds to the case of a short-embedded length (where the embedded length is less than the fully established softening length, $l_{sf,u}$). Equation (6) can be utilized to calculate the pull-out load at the end of the elastic phase. The solution for the elastic–softening stage can be obtained using Equations (7)–(10). The situation of the F-S relationship is different from the long bond length case (Figure 10a), as the shear stress distribution at the end of the elastic–softening stage is shown in Figure 11b stage III. The elastic and debonding regions cannot coexist for any value of the slip at the free end; i.e., before the shear stress at the loaded end drops to $\tau_{f}$, the shear stress at the free end reaches $\tau_{m}$. In the F-S curve, comparing Figure 10a or Figure 11a, it should be observed that the load decreases just after stage III for a short bond length. Besides, no snap-back phenomenon is observed. During the softening stage, all shear stresses along the entire embedded length fall within the softening branch. The distribution of shear stress along the bonded length is characterized by the second part of Equation (5), and the F-S relationship can be obtained as:

(20) $$S=-\frac{\lambda_{3}P}{\tau_{m}b}\frac{\left(s_{f}-s_{m}\right)}{\left(1-\mu \right)}\frac{1}{\tan\left(\lambda_{3}L\right)}+\frac{s_{f}-\mu s_{m}}{1-\mu }$$

When $S>s_{F}$, the interface enters the softening–debonding stage (see Stage IV in Figure 11b). The F-S relationship for this stage can be determined by Equations (15)–(18). Equation 19 can be used to compute the pull-out load at the end of the debonding stage (see Stage V in Figure 11b).

### 4.3. Verification and Determination of the Trilinear Bond–Slip Relationship

**Figure 12:**
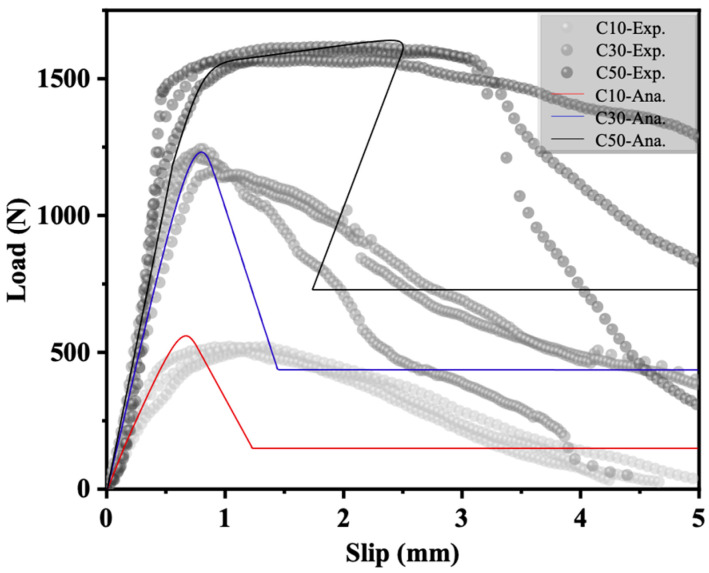
Comparison between experimental and analytical curves.

In this section, a trilinear bond–slip relationship (see Table 6) is employed in the analytical solutions presented in Section 4.2 and compared with the corresponding experimental load responses. Figure 12 compares the experimental and analytical F-S responses of the slip-out specimens. The experimental results and the analytical solution exhibit a slight dispersion; however, good consistency can be observed in the ascending region.

The snap-back phenomenon predicted by the analytical solution is distinct from the post-peak behavior observed in the experimental tests. This difference can be attributed to the control mode used for obtaining the experimental and analytical responses. In the analytical solution, the post-peak behavior was driven by the increase in $s_{F}$, which enables the development of the snap-back process, whereas the experimental tests were controlled by monotonically increasing the displacement (∆). This phenomenon is consistent with the explanation provided by Wu et al. [49] regarding the snap-back phenomenon of interfacial bond strength in external bonded reinforcement. In Wu et al.’s study [49], it was clearly pointed out that there exists a critical bond length that determines whether the analytical solution will exhibit a snap-back phenomenon (it occurs when the actual bond length exceeds this critical value and not when it is less than this value). The bond length mentioned here is the $l_{sf,u}$ defined in Section 4.2.

In addition, it can be observed from Figure 12 that the analytical responses obtained by using the trilinear bond–slip relationship model exhibit rounding at the peak of the given plot. Although the exact trilinear bond–slip relationship of embedded FRP strip–ECC joints cannot be precisely defined, the comparison results in Figure 12 indicate that it is reasonable to evaluate the parameters listed in Table 6.

## 5. Conclusions

In this study, the pull-out behavior of embedded CFRP strips in ECC was profoundly investigated through bench-scale tests. The effects of embedded lengths on interfacial bond strength and failure mode were comprehensively analyzed. An analytical method, based on a trilinear bond–slip relationship, for predicting the full-range behavior of embedded CFRP strip–ECC joints was introduced. Furthermore, the four key parameters of the trilinear bond–slip relationship of CFRP strip–ECC joints were identified. Several major findings are summarized as follows:

1. The epoxy resin remains adhered within the ECC matrix upon the pull-out of the FRP strip, indicating that the embedded FRP strip–ECC joint failure predominantly arises from interlayer shear damage occurring between the fibers and epoxy resin.
2. The *F*-*S* behavior for long embedded lengths includes an elastic stage, an elastic-softening stage, an elastic–softening–debonding stage, a softening–debonding stage, and a debonding stage. When the embedded length is shorter than the fully established softening length, the third stage becomes a pure softening stage.
3. The theoretical model introduced in this paper can accurately evaluate the pull-out behavior of embedded FRP strips in an ECC. However, the analytical model cannot guarantee accurate consistency with the post-peak behavior of the experimental results, especially when the embedded length is relatively long.

Through this study, it was found that FRP and ECC have excellent bonding properties. The failure of the FRP strip–ECC interface comes from the interlayer shear of the epoxy resin between the FRP and ECC. For the situation related to FRP and ECC in practical engineering, we should pay attention to the optimal selection and design of epoxy resin properties.

## Figures and Tables

**Table 1:** Mixture proportions of the engineered cementitious composite (ECC) (kg/m^3^).

Water	Cement	Fly Ash	Silica Sand	PE Fiber	Superplasticizer
400.00	533.30	400.00	266.60	15.00	4.00

**Table 2:** Chemical composition of fly ash and cement used (%).

	SiO_2_	Al_2_O_3_	Fe_2_O_3_	CaO	Others
Fly ash	45.20	26.10	6.93	11.84	9.93
Cement	17.94	4.46	3.56	64.56	9.48

**Table 3:** PE fiber properties.

Fiber Length	Diameter	Young’s Modulus	Elongation	Tensile Strength	Density
**(mm)**	**(μm)**	**(GPa)**	**(%)**	**(MPa)**	**(g/cm^3^)**
18	20	100	3	3000	0.97

**Table 4:** FRP properties (Reproduced from [36], Elsevier, 2023).

Type	Width	Thickness	Ultimate TensileStrength	Rupture Strain	Elastic Modulus
	(mm)	(mm)	(MPa)	(%)	(GPa)
CFRP	5.4	0.167	2462.96	1.10	218.79

**Table 5:** Details of test specimens.

Specimens ID	FRP Type	Embedded Length (mm)	Number	Failure Mode
C10	CFRP strip	10	3	Slid out
C30	CFRP strip	30	3	Slid out
C50	CFRP strip	50	3	Slid out
C70	CFRP strip	70	2	FRP rupture

**Table 6:** Parameters of the trilinear bond–slip relationships considered.

$s_{m}$ (mm)	$\tau_{m}$ (MPa)	$s_{f}$ (mm)	$\tau_{f}$ (MPa)
0.201	5.19	1.305	1.71

## Data Availability

The original contributions presented in this study are included in the article. Further inquiries can be directed to the corresponding author(s).
